# Prevalence of Sleep Apnea and Sleepiness in Adults With and Without HIV in Mwanza, Tanzania: Baseline Results From an Ongoing Cohort Study

**DOI:** 10.1111/jsr.70233

**Published:** 2025-10-30

**Authors:** Godfrey A. Kisigo, Benson Issarow, Salama Fadhil, Grace Ruselu, Ponsiano Fabian, Ayubu Garbindi, Robert N. Peck, Kathy Baisley, Saidi Kapiga, Ana C. Krieger

**Affiliations:** ^1^ London School of Hygiene and Tropical Medicine London UK; ^2^ Mwanza Intervention Trials Unit, National Institute for Medical Research Mwanza Tanzania; ^3^ Center for Global Health, Department of Medicine Weill Cornell Medicine New York New York USA; ^4^ Department of Medicine Weill Bugando School of Medicine Mwanza Tanzania; ^5^ Center for Sleep Medicine, Departments of Medicine and Neurology Weill Cornell Medical College New York New York USA

**Keywords:** epidemiology, HIV, Mwanza HIV&CVD cohort, objective measures, sleep apnea, sleepiness

## Abstract

We conducted a cross‐sectional analysis of the baseline survey of participants aged > 30 years enrolled in the Mwanza HIV&CVD Cohort in Tanzania. Our primary objective was to examine the association between HIV status and sleep apnea (SA). Secondary objectives were (1) to examine the association between HIV status and excessive daytime sleepiness (EDS) and (2) to identify risk factors associated with SA and with EDS. The cohort enrolled 500 people living with HIV (PLWH) and 500 people without HIV (PWoH) in 2021–2023. Participants completed overnight oximetry; SA was defined as an oxygen desaturation index (ODI) of ≥ 5 events/h. EDS was defined as an Epworth Sleepiness Scale score (ESS) of ≥ 11. The median age was 46 and 43 in PLWH and PWoH, respectively. The prevalence of sleep apnea was similar between PLWH and PWoH (17% and 19%, respectively; adjusted odds ratio (aOR) = 0.84, 95% confidence interval (CI) = 0.60–1.17). In contrast, the prevalence of EDS (ESS score ≥ 11) was higher in PWoH (21%) than in PLWH (13%) (aOR = 0.58, 95% CI = 0.41–0.83). In a multivariable model, factors associated with SA were older age, alcohol use, higher BMI category, hypertension and depression. Both objectively measured SA and subjectively reported EDS are common in Tanzanian adults. SA was strongly associated with overweight or obesity, suggesting that the prevalence of SA will grow with projected increases in age and obesity rates in Tanzania.

## 1 Background

Sleep apnea is characterised by recurrent episodes of abnormal breathing during sleep (Karna et al. 2023). Individuals with sleep apnea experience interrupted sleep episodes which may result in symptoms such as daytime sleepiness, fatigue and reduced cognitive function (Adams et al. 2001; Young et al. 1993). Clinically, sleep apnea presenting with symptoms is termed obstructive sleep apnea syndrome (Spicuzza et al. 2015). Obstructive sleep apnea syndrome has been associated with increased risk for cardiovascular disease, stroke and all‐cause mortality (Javaheri et al. 2024; Zhang et al. 2020). People living with HIV (PLWH) may be at high risk of developing sleep apnea due to several reasons. HIV‐associated lipohypertrophy is associated with fat deposition around the airway, affecting pharyngeal mechanics and causing sleep apnea (Lo Re et al. 2006; Carr, Samaras, Burton, et al. 1998; Carr, Samaras, Chisholm, and Cooper 1998). Furthermore, HIV may impair upper airway neural control, which in turn may worsen sleep apnea (Orr et al. 2021). HIV infection might also cause neuromuscular dysfunction and/or instability in ventilatory control, which has an additive effect on sleep apnea (Darquenne et al. 2015). Additionally, untreated sleep apnea could impact care engagement, antiretroviral therapy adherence and health outcomes among PLWH.

Most studies to assess the burden of sleep apnea among PLWH compared to people without HIV (PWoH) have been performed in high‐income countries and have reported inconclusive results (Patil et al. 2014; Punjabi et al. 2023; Kunisaki et al. 2021). However, two‐thirds of the world's PLWH population live in sub‐Saharan Africa (SSA), and do not have access to the same resources for testing or treating sleep apnea. According to a recent review, Africa had sleep laboratories in only four countries (29 in South Africa, 6 in Egypt, 4 in Nigeria and 2 in Kenya), and none of the African countries offers a certified or specialised sleep training program. Furthermore, the same review reported the association of HIV with sleep disorders, referencing only one study that reported sleep disorders in PLWH (Komolafe et al. 2021). Thus, very little is known about the burden of sleep apnea and its association with HIV in SSA, despite its relatively large population of PLWH.

Critically important, another major gap in the current sleep apnea literature is the lack of studies evaluating objective measures of sleep apnea in SSA (Wachinou et al. 2022). The majority of published studies used only subjective measures of sleep apnea (Komolafe et al. 2021; Ouédraogo et al. 2019; Sogebi et al. 2011; Jniene et al. 2012), which lack specificity and tend to underestimate the burden of sleep apnea (Chiu et al. 2017). Therefore, the current study used objective measures of overnight oximetry, along with subjective reports of sleepiness, in the Mwanza HIV&CVD Cohort to examine the association between HIV status and sleep apnea. Secondary objectives were (1) to examine the association between HIV status and sleepiness and (2) to identify risk factors associated with sleep apnea or sleepiness.

## 2 Methods

### 2.1 Overview

This cross‐sectional study involved all 1000 participants (500 PLWH and 500 PWoH) enrolled in the Mwanza HIV&CVD Cohort. Study participants were enrolled at the outpatient HIV clinic of Bugando Medical Centre (BMC) in Mwanza, the second largest city in Tanzania with an estimated population of approximately 1.1 million. The prevalence of HIV in the BMC catchment area is approximately 6%, which is similar to the national average (Tanzania Commission for AIDS (TACAIDS) 2024). The study analysed data from the baseline surveys which were completed by participants in the cohort between September 2021 and April 2023. The study received ethical approval from the Tanzanian National Institute for Medical Research [Ref: NIMR/HQ/R.8a/Vol. IX/4713], London School of Hygiene and Tropical Medicine [Ref: 30550] and Weill Cornell Medicine [Ref: 1506016328–21]. Study method and results are reported following the Strengthening the Reporting of Observational Studies in Epidemiology (STROBE) Statement for cross‐sectional studies (von Elm et al. 2007).

### 2.2 Study Participants

Participants in the Mwanza HIV&CVD Cohort were recruited from the same HIV care outpatient clinic. PLWH and PWoH who attended the clinic during the study period were approached separately by the study research nurse in the clinic waiting area and invited to learn more about the study. Those who expressed interest were referred to the study team for eligibility screening and consent procedures. Individuals were eligible to be enrolled if they were aged ≥ 30 years and able and willing to provide written informed consent. Additionally, PLWH were required to be on antiretroviral (ART) medications ≥ 180 days prior to study entry. PWoH were required to be a ‘treatment supporter’ of an adult attending the clinic. HIV treatment supporters are close friends or relatives of PLWH named as potential contacts and sources of support when an individual enrolls in HIV care (NACP‐National AIDS Control Programme 2019). We have previously shown that treatment supporters have sociodemographic characteristics similar to those of PLWH in Tanzania since they are drawn from the same source population (Reis et al. 2020). No attempt was made to recruit PLWH and their treatment supporters as matched pairs; however, according to the principles of frequency matching, we monitored recruitment to ensure balanced enrollment by sex and age group. Treatment supporters could be enrolled even if the PLWH whom they were supporting was not enrolled in the study.

### 2.3 Sample Size

Our study sample size was fixed by the size of the Mwanza HIV&CVD Cohort (*N* = 1000). With 500 PLWH and 500 PWoH, we had 80% power to detect an increase in prevalence from 20% to 27.5% (38% relative increase) or from 40% to 48.8% (22% relative increase). With a sample size of 1000, assuming an overall prevalence of sleep apnea of 20%, the prevalence of risk factors among people without sleep apnea to be between 20% and 40%, we would have 80% power to detect an odds ratio (OR) of ≥ 1.65 for the association between the risk factor and sleep apnea. If prevalence was 40%, we would have 80% power to detect an OR of ≥ 1.50.

### 2.4 Sleep Data

#### 2.4.1 Sleepiness

The Epworth Sleepiness Scale (ESS) was used to measure excessive daytime sleepiness (Johns 1991). The ESS has been adapted for the East African context, translated into Kiswahili (the national language of Tanzania), and used in several previous studies in Tanzania (Shayo and Mugusi 2015; Kundy 2016). The ESS includes eight items that explore the usual chances of dozing off or falling asleep while engaged in eight different activities. Each item was answered on a 4‐point scale, from 0 (would never doze) to 3 (high chance of dozing). The items were summed, with a possible score range of 0–24. ESS scores of ≥ 11 represented excessive daytime sleepiness (Johns 1993; Sanford et al. 2006).

#### 2.4.2 Sleep Apnea

The overnight oxygen saturation was measured using the Nonin WristOx2 Model 3150 wrist‐worn pulse oximeter with soft sensor clips at a 4‐s sampling rate. Overnight oximetry reports were generated using Nonin's Nvision software (v6.4). Each recording was examined for quality check, and a successful measurement was at least 3 h of continuous high‐quality oximetry data. Desaturation events were determined based on a drop in arterial oxygen saturation by at least 4% from a local baseline for a minimum duration of 10 s. Sleep apnea was defined as an oxygen desaturation index (ODI) of ≥ 5 per hour, with ODI determined by the number of desaturation events per hour (Gumb et al. 2018). Sleep apnea can result from two types of events: airway obstruction events, known as obstructive sleep apnea (OSA), and central respiratory drive events, referred to as central sleep apnea (CSA). However, overnight oximetry does not differentiate between these two types of events. Therefore, the current study uses the broader term ‘sleep apnea’ to describe the condition.

#### 2.4.3 Demographic and Clinical Data

Participants completed a standardised questionnaire and underwent a physical examination by trained staff at enrollment. The standardised physical examination, including measurements for weight and height, was performed according to the World Health Organization's STEPwise Surveillance protocol (World Health Organization (WHO) 2002). For PLWH, CD4+ T‐cell count was measured using an automated BD FACSCalibur System (BD Biosciences).

### 2.5 Statistical Analysis

Analyses were conducted in Stata 18 (StataCorp LLC, College Station, TX). Baseline demographic and clinical characteristics were summarised by median and interquartile range (IQR) for continuous variables and frequency and percentages for categorical variables. We compared the prevalence of sleep apnea, and of excessive daytime sleepiness, between PLWH and PWoH using Chi‐square tests. We used logistic regression to estimate ORs and 95% confidence intervals (CI) for the association of HIV infection with sleep apnea, and with excessive daytime sleepiness, adjusted for potential confounders. Potential confounders were pre‐specified a priori based on known risk factors for sleep apnea, and for excessive daytime sleepiness, that were not thought to be on the causal pathway between HIV status and the outcome. For sleep apnea, these were age group (30–39, 40–49, ≥ 50 years), sex, alcohol use, and smoking. For excessive daytime sleepiness, these were age group, sex, education, marital status, socioeconomic status and alcohol use. As a sensitivity analysis, we also included BMI in the models as a potential confounder. All potential confounders were fitted in the models as categorical terms.

We examined risk factors associated with sleep apnea or excessive daytime sleepiness, using a conceptual hierarchical framework with three levels: sociodemographic factors (distal determinants), behavioural factors and clinical factors and comorbidities (proximal determinants) (Victora et al. 1997). Age group and sex were considered a priori confounders and included in all models. First, sociodemographic factors whose age‐ and sex‐adjusted association with the outcome was significant at *p* < 0.10 were included in a multivariable logistic regression model; those remaining associated at *p* < 0.10 were retained in a ‘core’ sociodemographic model. Next behavioural variables were added to this core model one by one and retained if they remained associated at *p* < 0.10. Associations with clinical and comorbidity variables were assessed in a similar way, adjusted for sociodemographic and behavioural factors. All covariates were fit as categorical factors.

To evaluate whether the association of risk factors with each sleep outcome was modified by HIV status, we assessed effect modification by including an interaction term between HIV status and the covariates in the final regression models at each hierarchical level. Potential effect modification with each covariate was tested in a separate model.

## 3 Results

### 3.1 Description of Participants

A total of 1000 participants (500 PLWH and 500 PWoH) were enrolled in the study and completed the baseline survey. Of the 1000 participants who completed the overnight oxygen saturation assessment, 99% (499 PLWH and 497 PWoH) of the records passed quality checks and were included in the analysis. The median age [IQR] was similar between the two groups with 46 [39–50] and 43 [36–49] years in PLWH and PWoH, respectively. Overweight and obesity were slightly more common in PWoH than in PLWH (26% vs. 21%, 15% vs. 13%, respectively). In PLWH, the median CD4 cell count was 716 cells/mL, reflecting well‐controlled disease. All PLWH were on a combination of dolutegravir (an integrase strand‐transfer inhibitor) and two nucleoside reverse‐transcriptase inhibitors, namely tenofovir and lamivudine. The detailed demographic characteristics of the study participants are presented in Table 1.

**TABLE 1:** Socio‐demographic characteristics among participants in the Mwanza HIV&HTN Sleep Cohort baseline survey.

Variable	Total (*N* = 1000)	PLWH (*n* = 500)	PWoH (*n* = 500)
Sex			
Male	302 (30%)	149 (30%)	153 (31%)
Female	698 (70%)	351 (70%)	347 (69%)
Age in years (median, IQR)	44 (38–50)	46 (39–50)	43 (36–49)
Education level			
No/incomplete primary	190 (19%)	109 (22%)	81 (16%)
Primary school education	617 (62%)	318 (64%)	299 (60%)
Secondary/college/University	193 (19%)	73 (14%)	120 (24%)
Marital status			
Married/cohabiting	598 (60%)	238 (48%)	360 (72%)
Divorced/separated	217 (22%)	149 (30%)	68 (14%)
Widowed	144 (14%)	96 (19%)	48 (9%)
Single	41 (4%)	17 (3%)	24 (5%)
Socioeconomic status score^a^ (median, IQR)	2 (0–3)	2 (0–3)	2 (1–3)
Score 0	251 (25%)	143 (28%)	108 (22%)
Score 1	161 (16%)	84 (17%)	77 (15%)
Score 2	231 (23%)	105 (21%)	126 (25%)
Score ≥ 3	357 (36%)	168 (34%)	189 (38%)
Current alcohol use			
Yes	299 (30%)	153 (31%)	146 (29%)
Current tobacco smoking			
Yes	57 (6%)	20 (4%)	37 (7%)
Probable depression^b^			
Absent	888 (89%)	437 (87%)	451 (90%)
Present	112 (11%)	63 (13%)	49 (10%)
Body Mass Index (BMI) (Kg/m^2^) (median, IQR)	23.1 (20.2–27.1)	22.7 (20.0–26.5)	23.4 (20.6–27.2)
Underweight < 18.5	91 (9%)	49 (10%)	42 (9%)
Normal 18.5–24.9	539 (54%)	283 (56%)	256 (51%)
Overweight 25.0–29.9	234 (23%)	103 (21%)	131 (26%)
Obese ≥ 30	136 (14%)	65 (13%)	71 (14%)
CD4 count (cells/mL)	801 (623–1007)	716 (537–953)	851 (706–1047)
Comorbidities			
Hypertension^c^	114 (11%)	59 (12%)	55 (11%)
Diabetes mellitus^d^	30 (3%)	17 (3%)	13 (3%)
Chronic kidney disease^e^	38 (4%)	33 (7%)	5 (1%)

Abbreviations: PLWH, people living with HIV; PWoH, people without HIV.

^a^ A composite score combining ownership of four household assets (television, refrigerator, motorcycle and car) and presence of tap water and electricity, each contributing a 1‐point.

^b^ Patient Health Questionnaire (PHQ‐9) score of ≥ 10.

^c^ Clinic systolic blood pressure ≥ 140 or diastolic blood pressure ≥ 90.

^d^ Fasting blood glucose ≥ 7.0 mmol/L.

^e^ Estimated glomerular filtration rate (eGFR) < 60 mL/min/1.73 m^2^.

### 3.2 Prevalence of Sleep Apnea and Excessive Daytime Sleepiness and Association With HIV

The prevalence of sleep apnea was similar between PLWH and PWoH (17% and 19%, respectively; *p* = 0.436). There was no evidence of an association of HIV status with sleep apnea, after adjusting for potential confounders (adjusted odds ratio (aOR) = 0.84; 95% confidence interval (CI) = 0.60–1.17; Table 2). Interestingly, the prevalence of excessive daytime sleepiness was lower in PLWH (13%) than in PWoH (21%) (*p* = 0.001). After adjusting for potential confounders, PLWH remained with a lower odds of excessive daytime sleepiness when compared to PWoH (aOR = 0.58, 95% CI: 0.41–0.83). Only 29 (out of 998) participants (PLWH, *n* = 11; PWoH, *n* = 18) had both sleep apnea and excessive daytime sleepiness, thus meeting the definition of obstructive sleep apnea syndrome. The results from the sensitivity analysis accounting for potential confounding by BMI of the association of HIV with sleep apnea and excessive daytime sleepiness were similar to those of the primary analysis (Table S1).

**TABLE 2:** Prevalence and association of sleep apnea and excessive daytime sleepiness with HIV status.

	Prevalence	Crude analysis		Adjusted analysis	
	*n* (%)	OR [95% CI]	*p*	aOR [95% CI]	*p*
Sleep apnea					
PWoH	92/497 (19%)	1		1	
PLWH	83/499 (17%)	0.88 [0.63–1.22]	0.436	0.84 [0.60–1.17]^a^	0.296
Excessive daytime sleepiness					
PWoH	104/500 (21%)	1	0.001	1	0.003
PLWH	64/500 (13%)	0.56 [0.40–0.79]		0.58 [0.41–0.83]^b^	

Abbreviations: PLWH, people living with HIV; PWoH, people without HIV.

^a^ Adjusted for age group (30–39, 40–49, ≥ 50 years), sex, alcohol use and smoking.

^b^ Adjusted for age group, sex, education, marital status, socioeconomic status and alcohol use.

### 3.3 Factors Associated With Sleep Apnea

**FIGURE 1:**
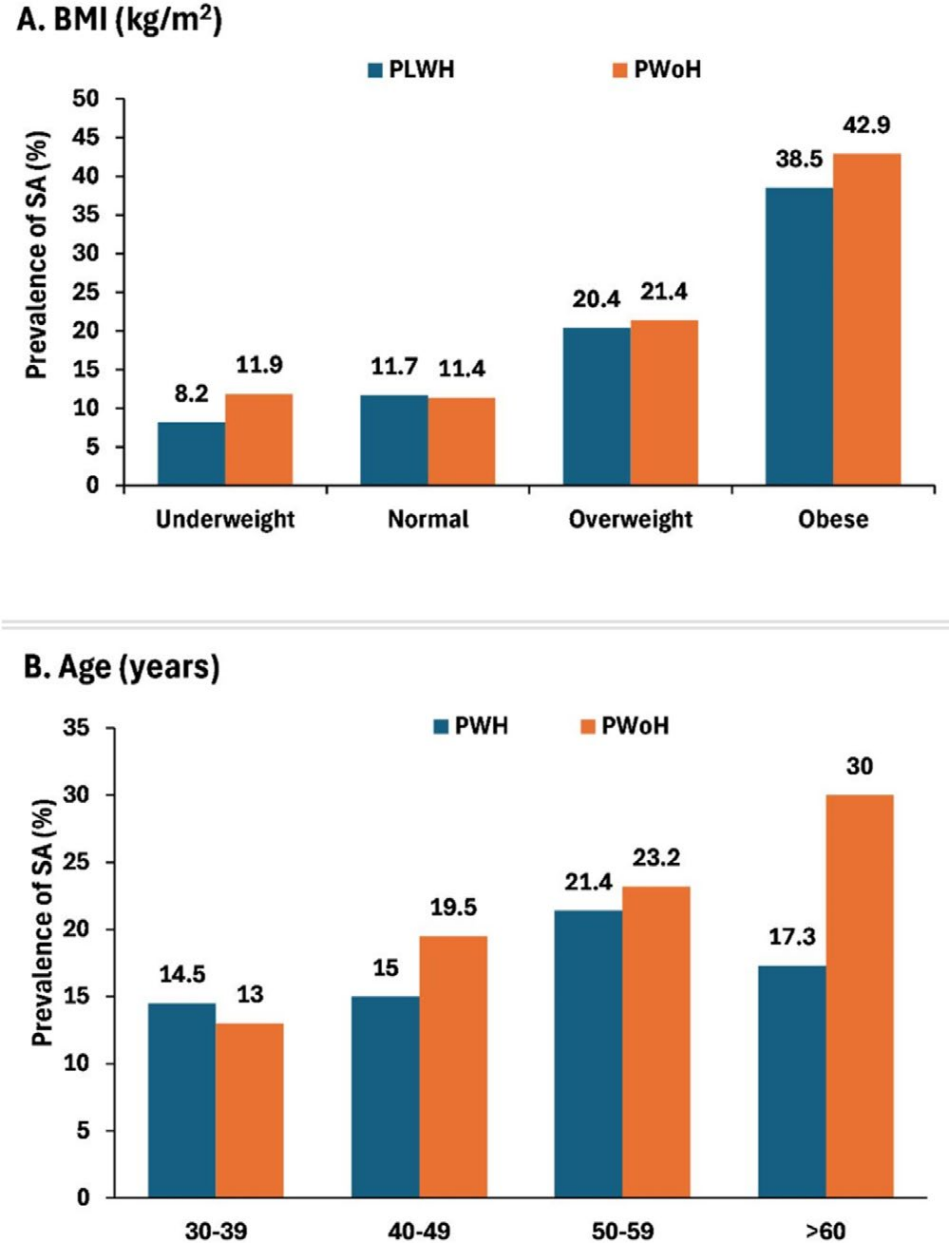
Prevalence of sleep apnea by body mass index (BMI) (A) and age (B).

The prevalence of sleep apnea according to BMI and age categories is shown in Figure 1. There was evidence that the prevalence of sleep apnea increased with age, but there was no evidence of an association with other sociodemographic factors (Table 3). After adjusting for age and sex, there was some evidence of an association with alcohol use (aOR = 1.38, 95% CI = 0.97–1.96). After adjusting for age, sex and alcohol use, there was strong evidence of an association with BMI (aOR = 2.20 [95% CI = 1.44–3.37] and aOR = 6.48 [95% CI = 4.05–10.37] comparing overweight and obese, respectively, with normal/underweight). There was also strong evidence of an association with hypertension (aOR = 2.03, 95% CI = 1.26–3.25) and some evidence of an association with depression (aOR = 1.73, 95% CI = 1.02–2.91). There was no evidence of an association with other clinical factors or comorbidities.

**TABLE 3:** Factors associated with sleep apnea.

	*n* with sleep apnea/*N* (%)	Unadjusted odds ratio [95% CI]	Adjusted odds ratio^a^ [95% CI]
*Sociodemographic factors*			
Age category (years)		*p* = 0.018	*p* = 0.018
30–< 40	41/301 (14%)	1	1
40–< 50	72/421 (17%)	1.31 [0.86–1.98]	1.31 [0.86–1.98]
50 and above	62/274 (23%)	**1.85 [1.20–2.86]**	**1.86 [1.20–2.87]**
Sex		*p* = 0.840	*p* = 0.97
Females	121/695 (17%)	1	1
Males	54/301 (18%)	1.04 [0.73–1.48]	0.99 [0.70–1.42]
Education level		*p* = 0.153	*p* = 0.25
No/incomplete primary	35/190 (18%)	1	1
Primary school education	115/613 (19%)	1.02 [0.67–1.56]	1.03 [0.67–1.58]
Secondary/college/University	25/193 (13%)	0.66 [0.38–1.15]	0.70 [0.39–1.24]
Marital status		*p* = 0.771	*p* = 0.93
Divorced/widowed/single	72/400 (18%)	1	1
Married/cohabiting	103/596 (17%)	0.95 [0.68–1.33]	0.98 [0.69–1.39]
Socioeconomic status^b^		*p* = 0.507	*p* = 0.40
Score 0	37/250 (15%)	1	1
Score 1	28/160 (17%)	1.22 [0.71–2.09]	1.25 [0.73–2.14]
Score 2	46/230 (20%)	1.44 [0.89–2.32]	1.51 [0.94–2.44]
Score ≥ 3	64/356 (18%)	1.26 [0.81–1.96]	1.27 [0.82–1.99]
*Behavioural factors*			
Alcohol use		*p* = 0.106	*p* = 0.08
No	114/700 (16%)	1	1
Yes	61/296 (21%)	1.33 [0.94–1.89]	1.38 [0.97–1.96]
Current smoking		*p* = 0.487	*p* = 0.66
No	163/939 (17%)	1	1
Yes	12/57 (21%)	1.27 [0.66–2.45]	1.18 [0.57–2.42]
*Clinical factors and comorbidities*			
Body mass index (kg/m^2^)		*p* < 0.001	*p* < 0.001
< 25	71/627 (11%)	1	1
25–29.99	49/234 (21%)	**2.07 [1.39–3.09]**	**2.20 [1.44–3.37]**
≥ 30	55/135 (41%)	**5.38 [3.53–8.22]**	**6.48 [4.05–10.37]**
Hypertension^c^		*p* < 0.001	*p* = 0.004
Absent	141/882 (16%)	1	1
Present	34/114 (30%)	**2.23 [1.44–3.47]**	**2.03 [1.26–3.25]**
Diabetes mellitus^d^		*p* = 0.727	*p* = 0.43
Absent	169/966 (17%)	1	1
Present	6/30 (20%)	1.18 [0.47–2.93]	0.68 [0.26–1.82]
Chronic kidney disease^e^		*p* = 0.889	*p* = 0.66
Absent	168/958 (18%)	1	1
Present	7/38 (18%)	1.06 [0.46–2.45]	0.82 [0.34–2.00]
Probable depression^f^		*p* = 0.364	*p* = 0.047
Absent	152/885 (17%)	1	1
Present	23/111 (21%)	1.26 [0.77–2.06]	**1.73 [1.02–2.91]**
HIV status^g^		*p* = 0.436	*p* = 0.48
PWoH	92/497 (19%)	1	1
PLWH	83/499 (17%)	0.88 [0.63–1.22]	0.88 [0.62–1.25]

^a^ Sociodemographic factors are adjusted for age group (30–39, 40–49, ≥ 50 years) and sex. Behavioural factors are adjusted for age group, sex and alcohol use. Clinical factors and comorbidities are adjusted for age group, sex, alcohol use, body mass index and hypertension.

^b^ A composite score combining ownership of four household assets (television, refrigerator, motorcycle and car) and presence of tap water and electricity, each contributing a 1‐point.

^c^ Patient Health Questionnaire (PHQ‐9) score of ≥ 10.

^d^ Clinic Systolic blood pressure ≥ 140 or diastolic blood pressure ≥ 90.

^e^ Fasting blood glucose ≥ 7.0 mmol/L.

^f^ Estimated Glomerular Filtration Rate (eGFR) < 60 mL/min/1.73 m^2^.

^g^ PLWH, people living with HIV; PWoH, people without HIV.

For most risk factors, there was no evidence that the association with sleep apnea differed by HIV status (interaction *p*‐values ≥ 0.58). However, there was some evidence of effect modification between HIV status and alcohol use, with a higher odds of sleep apnea among those who drank alcohol in PLWH, but not PWoH (interaction *p*‐value = 0.04; Table S2). There was also weak evidence of effect modification between HIV status and hypertension (*p* = 0.11).

### 3.4 Factors Associated With Excessive Daytime Sleepiness

After adjusting for age and sex, there was evidence that people who were married or living with their partner had higher odds of excessive daytime sleepiness (aOR = 1.64, 95% CI = 1.14–2.37), but there was no evidence of an association with other sociodemographic factors (Table S3). After adjusting for age, sex and marital status, there was some evidence of an association with alcohol use (aOR = 1.57, 95% CI = 1.09–2.24). After adjusting for sociodemographic and behavioural factors, there was strong evidence of an association with depression (aOR = 1.99, 95% CI = 1.23–3.20). There was also strong evidence of an association with HIV status, with PLWH having lower odds of excessive daytime sleepiness (aOR = 0.57, 95% CI = 0.40–0.81). There was no evidence of an association with other clinical factors or comorbidities.

There was no evidence that the association of most risk factors with excessive daytime sleepiness differed by HIV status (interaction *p*‐value ≥ 0.17). However, there was some evidence that the association with depression differed by HIV status (*p*‐value for interaction = 0.03), with a higher odds of excessive daytime sleepiness among those with depression in PLWH, but not PWoH (Table S4).

## 4 Discussion

In this large cross‐sectional study, we are among the first to report the prevalence of objectively measured sleep apnea in SSA and the first from East Africa. We found that sleep apnea was common in our cohort, and its prevalence was similar between PLWH and PWoH. Excessive daytime sleepiness, as measured by the Epworth sleepiness scale, was also common; however, it was less prevalent in PLWH than in PWoH. We also found a higher prevalence of sleep apnea in people with higher BMI, independent of HIV status. This corroborates with the literature findings that overweight and obesity are risk factors for sleep apnea. Additionally, our findings highlight a significant public health concern given that the prevalence of overweight and obesity among the Tanzanian population is increasing (Keino and Carrel 2023). These findings underline the importance of building capacity for the diagnosis and treatment of sleep apnea and weight management in SSA.

Any generalisation of the results from our study must be done with caution, even for populations within SSA. The participants of the Mwanza HIV&CVD Cohort are representative of HIV‐infected persons in urban Tanzania with well‐controlled disease on ART. Thus, our findings may not apply to rural‐dwelling adults or people with untreated HIV infection. Moreover, we recruited participants from among interested individuals attending the outpatient HIV clinic during the study period, which may have introduced selection bias. Treatment supporters selected as controls might be more likely to experience excessive daytime sleepiness than the general population, which may have biased our estimate away from the null and explain our finding of an apparent ‘protective’ effect of HIV. Additionally, our findings are based on cross‐sectional data, which limits our ability to determine causality. The identified associations should not be interpreted as a causal relationship and directionality cannot be assumed. Also, as in any observational study, the adjusted estimates may have been affected by residual confounding, owing to unmeasured factors, or imperfect measurement or modelling of included factors. Although we adjusted for age category, the age bands were quite wide so the adjustment may have been imperfect. However, the age distribution was similar in PLHIV and PWoH, so this is unlikely to have a large impact on our findings. The use of overnight oximetry to assess sleep apnea might have slightly overestimated its prevalence due to the dark skin pigmentation of our study population. However, the fingertip pulse oximeter used in the current study has been reported to perform better in dark‐pigmented individuals, and any potential measurement bias would not be different between groups (Leeb et al. 2024).

The current study is among the few studies that objectively investigated the prevalence of sleep apnea in SSA. To the best of our knowledge, we are the first researchers to conduct overnight oximetry assessment in a large sample of adults in the eastern Africa region to evaluate for sleep apnea. We found that the overall prevalence of sleep apnea was 17.6%. This prevalence is somewhat low compared to the previous studies conducted in Western Africa, which ranged from 40.2% to 43.3% (Wachinou et al. 2022; Massongo et al. 2020). This difference could be attributed to the younger age of our participants and the relatively low proportion of obesity in our cohort compared to the participants in those studies.

As far as we know, only three previous studies have objectively compared the prevalence of sleep apnea between people with and without HIV. Two of these studies were conducted within the Multicenter AIDS Cohort Study (MACS) in the United States (Patil et al. 2014; Punjabi et al. 2023), and the third was within the Pharmacokinetics and Clinical Observations in People Over Fifty (POPPY) Study in the United Kingdom (Kunisaki et al. 2021). Our finding of comparable prevalence of sleep apnea between PLWH and PWoH is consistent with that observed in the POPPY study, which showed no difference (7% vs. 8%) based on HIV status. Data on the prevalence of sleep apnea in men with HIV in the MACS study are conflicting. In an earlier analysis, men with HIV had a lower prevalence of sleep apnea compared to men without HIV (72% vs. 87%, respectively) (Patil et al. 2014). In contrast, the second MACS study reported a higher prevalence of sleep apnea in men with HIV compared to men without HIV (57% vs. 47%, respectively). The differences in these reports warrant further investigation into the putative pathophysiological mechanisms by which HIV affects sleep apnea.

It is interesting to note the strikingly higher prevalences of sleep apnea reported in both MACS studies compared to our cohort and POPPY studies. There are two possible explanations for this observation. First, the MACS used polysomnography, which allows the assessment of electroencephalogram (EEG) to detect arousal and total sleep time. The current study and POPPY study used overnight oximetry to determine the presence or absence of sleep apnea without concurrently using an EEG. As such, the quantification of sleep apnea did not include EEG arousals for scoring hypopneas, and time in bed was used instead of total sleep time. The differences in methods could have led to an underestimation of the prevalence of sleep apnea. Second, the demographics and clinical characteristics of the participants in the MACS study influenced the higher prevalence of sleep apnea. For example, only men were included in the MACS study, while the current and POPPY studies included both men and women. The men in the MACS study had a relatively higher body mass index (PLWH = 27.2 kg/m^2^, men without HIV = 27.9 kg/m^2^) than participants in both current (PLWH = 22.6 kg/m^2^, community controls = 23.5 kg/m^2^) and POPPY (PLWH = 25.6 kg/m^2^, controls = 26.0 kg/m^2^) studies. It has already been established that male sex and overweight are risk factors for sleep apnea. Therefore, these differences in the study population characteristics likely influenced the results.

In this study, PLWH were less likely to report excessive daytime sleepiness than PWoH. This finding was unexpected given the hypothesis that HIV affects the continuity of nocturnal sleep, resulting in excessive daytime sleepiness (Robbins et al. 2004; Norman 1988). As such, we anticipated PLWH to experience more significant daytime sleepiness. While it is difficult to explain this result, perhaps the benefit of being in an HIV primary care clinic, with regular health education about self‐care and well‐being might have introduced a bias. The effect of supportive care for chronic illness could have led to increased health literacy and a greater focus on living a healthy lifestyle, ultimately improving PLWH's daytime alertness.

We found that overweight and obese individuals are associated with sleep apnea, with no evidence that the association differed by HIV status. The association with BMI is in accordance with findings reported by previous studies in both high‐ and low‐income countries (Punjabi et al. 2023; Wachinou et al. 2022; Johnson et al. 2018). We also found that older age, alcohol use, hypertension and depression were associated with sleep apnea. There was also some evidence that the association with alcohol use and with hypertension differed by HIV status, although the confidence intervals were wide. Furthermore, this study was not powered to detect effect modification, so these findings should be interpreted as exploratory and with caution.

After adjusting for potential confounders, we found that PLWH had a lower odds of excessive daytime sleepiness than PWoH. We also found a positive association between excessive daytime sleepiness and being married/living with a partner, alcohol use and depression. There was some evidence that the association with depression differed by HIV status; however, this finding should be interpreted with caution.

As mentioned, both sleep apnea and excessive daytime sleepiness were relatively common in our cohort (overall prevalence of each outcome ~17%). Thus, our reported odds ratios for associations should not be interpreted as risk ratios. For example, the crude odds ratio for the association of HIV with excessive daytime sleepiness is 0.56, whereas the risk ratio is 0.62.

We have described the spectrum of sleep apnea in a Tanzanian population of people with and without HIV. In our study, the prevalence of Obstructive Sleep Apnea Syndrome (OSAS) was low with only 2.5% of participants experiencing both sleep apnea and excessive daytime sleepiness. The reason for this low prevalence of OSAS is not immediately apparent. It could be related to the underreporting of daytime sleepiness in our population due to language or cultural issues in applying the Epworth Sleepiness Scale to an East African population. On the other hand, this finding could indicate a high prevalence of sleep apnea that is truly not associated with excessive daytime sleepiness, but might or might not be associated with other medical complications. Further studies are needed to determine the real public health burden of OSAS in East Africa.

In summary, this cross‐sectional analysis suggests that sleep apnea and sleepiness are common in PLWH and PWoH in Mwanza, Tanzania. Clinical sleep apnea diagnostic and treatment services are lacking in many countries in SSA including Tanzania. Since sleep apnea notably impacts quality of life and cardiovascular conditions, improved capacity for sleep medicine services is urgently needed. Further studies using reference‐standard sleep studies incorporating respiratory polygraphy and EEG will be useful to further investigate sleep apnea in SSA.

## Author Contributions

All authors fulfil the criteria for authorship. Conceptualisation, methodology and data curation: G.A.K., R.N.P, S.K. and A.C.K. Investigation: G.A.K., B.I., S.F,. P.F., A.G., K.B., R.N.P, S.K. and A.C.K. Formal analysis: G.A.K., K.B., R.N.P., S.K. and A.C.K. Writing – original draft: G.A.K. Supervision: K.B., R.N.P., S.K. and A.C.K. Project administration: B.I., S.F., R.N.P. and S.K. Funding acquisition: R.N.P, S.K. and A.C.K. Resources: S.K. Visualization: K.B. Writing – review and editing: All authors.

## Disclosure

The authors have nothing to report.

## Conflicts of Interest

The authors declare no conflicts of interest.

## Supporting information


**Table S1:** Sensitivity analysis of association of sleep apnea and excessive daytime sleepiness with HIV status, including body mass index as a confounder.
**Table S2:** Factors associated with sleep apnea, stratified by HIV status^1^.
**Table S3:** Factors associated with excessive daytime sleepiness.
**Table S4:** Factors associated with excessive daytime sleepiness, stratified by HIV status^1^.

## Data Availability

The data that support the findings of this study are available on request from the corresponding author. The data are not publicly available due to privacy or ethical restrictions.
