# Supplementary material for: Prevalence of Sleep Apnea and Sleepiness in Adults With and Without HIV in Mwanza, Tanzania: Baseline Results From an Ongoing Cohort Study
Source: J Sleep Res. 2025 Oct 30;35(3):e70233. doi: 10.1111/jsr.70233 (PMC12771531; doi:10.1111/jsr.70233)
Supplement: Supplementary file 1 — Table S1: Sensitivity analysis of association of sleep apnea and excessive daytime sleepiness with HIV status, including body mass index as a confounder. Table S2: Factors associated with sleep apnea, stratified by HIV status1. Table S3: Factors associated with excessive daytime sleepiness. Table S4: Factors associated with excessive daytime sleepiness, stratified by HIV status1. [file JSR-35-e70233-s001.docx]

**Supplementary table 1**: Sensitivity analysis of association of sleep apnea and excessive daytime sleepiness with HIV status, including body mass index as a confounder

|  | **Prevalence** | **Crude Analysis** | | **Adjusted Analysis** | |
| --- | --- | --- | --- | --- | --- |
|  | **n (%)** | **OR [95% CI]** | **p-value** | **aOR [95% CI]** | **p-value** |
| Sleep apnea | | | | | |
| PWoH | 92 /497 (19%) | 1 |  | 1 |  |
| PLWH | 83 / 499 (17%) | 0.88 [0.63-1.22] | 0.436 | 0.91 [0.65-1.61]^1^ | 0.614 |
| Excessive daytime sleepiness | | | | | |
| PWoH | 104 /500 (21%) | 1 |  | 1 |  |
| PLWH | 64 / 500 (13%) | 0.56 [0.40-0.79] | 0.001 | 0.59 [0.41-0.84]^2^ | 0.004 |

PLWH: People Living with HIV, PWoH: People without HIV

^1^Adjusted for age group (30-39, 40-49, ≥50 years), sex, alcohol use, smoking, and body mass index. ^2^Adjusted for age group, sex, education, marital status, socioeconomic status, alcohol use, and body mass index.

**Supplementary table 2.** Factors associated with sleep apnea, stratified by HIV status^1^

|  | **Adjusted odds ratio [95% CI]** ^2^ | | **p-value for interaction**^1^ |
| --- | --- | --- | --- |
|  | **PWoH** | **PLWH** |  |
| **Sociodemographic** |  |  |  |
| Age category (years) |  |  | 0.576 |
| 30 - <40 | 1 | 1 |  |
| 40 - <50 | 1.62 (0.92 -2.85 ) | 1.04 (0.56 -1.94 ) |  |
| 50 and above | 2.21 (1.21 -4.01 ) | 1.55 (0.82 -2.93 ) |  |
| Sex |  |  | 0.654 |
| Females | 1 | 1 |  |
| Males | 0.92 (0.56 -1.51 ) | 1.08 (0.65 -1.80 ) |  |
| **Behavioral** |  |  |  |
| Alcohol use |  |  | 0.040 |
| No | 1 | 1 |  |
| Yes | 0.96 (0.57 -1.59 ) | 1.99 (1.21 -3.26 ) |  |
| **Clinical and comorbidities** |  |  |  |
| Body mass index (kg/m2) |  |  | 0.912 |
| <25 | 1 | 1 |  |
| 25-29.99 | 2.14 (1.21 -3.80 ) | 2.23 (1.21 -4.11 ) |  |
| ≥30 | 6.95 (3.69 -13.09) | 5.94 (3.12 -11.30) |  |
| Hypertension^3^ |  |  | 0.109 |
| Absent | 1 | 1 |  |
| Present | 2.92 (1.53 -5.56 ) | 1.36 (0.67 -2.74 ) |  |
| Probable depression^4^ |  |  | 0.941 |
| Absent | 1 | 1 |  |
| Present | 1.71 (0.79 -3.69 ) | 1.78 (0.88 -3.57 ) |  |

PLWH: People Living with HIV, PWoH: People without HIV

^1^Stratified estimates are obtained by fitting an interaction term between HIV status and each covariate, with p-values obtained by comparing models with and without the interaction term. ^2^Sociodemographic factors are adjusted for age group (30-39, 40-49, ≥50 years) and sex. Behavioral factors are adjusted for age group, sex and alcohol use. Clinical factors and comorbidities are adjusted for age group, sex, alcohol use, body mass index, and hypertension. ^3^Clinic Systolic blood pressure ≥140 or diastolic blood pressure ≥90. ^4^Patient Health Questionnaire (PHQ-9) score of ≥10.

**Supplementary table 3.** Factors associated with excessive daytime sleepiness

|  | **n with excessive daytime sleepiness / N (%)** | **Unadjusted odds ratio [95% CI]** | **Adjusted odds ratio**^1^ **[95% CI]** |
| --- | --- | --- | --- |
| **Sociodemographic** |  | | |
| Age category (years) |  | P=0.238 | P=0.16 |
| 30 - <40 | 46/303 (15%) | 1 | 1 |
| 40 - <50 | 67/423 (16%) | 1.05 [0.70-1.58] | 1.08 [0.71-1.62] |
| 50 and above | 55/274 (20%) | 1.40 [0.91-2.16] | 1.48 [0.96-2.30] |
| Sex |  | P=0.677 | P=0.62 |
| Females | 53/302 (18%) | 1 | 1 |
| Males | 115/698 (16%) | 1.08 [0.75-1.54] | 0.91 [0.63-1.32] |
| Education level |  | P=0.556 | P=0.54 |
| No/incomplete primary | 27/190 (14%) | 1 | 1 |
| Primary school education | 107/617 (17%) | 1.27 [0.80-2.00] | 1.27 [0.80-2.02] |
| Secondary /college/University | 34/193 (18%) | 1.29 [0.74-2.24] | 1.33 [0.76-2.36] |
| Marital status |  | P=0.011 | P=0.007 |
| Divorced/ Widowed/ Single | 53/402 (13%) | 1 | 1 |
| Married/cohabiting | 115/598 (19%) | **1.57 [1.10-2.23]** | **1.64 [1.14-2.37]** |
| Socioeconomic status^2^ |  | P=0.544 | P=0.48 |
| Score 0 | 49/251 (20%) | 1 | 1 |
| Score 1 | 23/161 (14%) | 0.69 [0.40-1.18] | 0.69 [0.40-1.20] |
| Score 2 | 38/231 (16%) | 0.81 [0.51-1.30] | 0.81 [0.50-1.29] |
| Score ≥3 | 58/357 (16%) | 0.80 [0.53-1.22] | 0.74 [0.48-1.14] |
| **Behavioral** |  | | |
| Alcohol use |  | P=0.032 | P=0.015 |
| No | 106/701 (15%) | 1 | 1 |
| Yes | 62/299 (21%) | **1.47 [1.04-2.08]** | **1.57 [1.09-2.24]** |
| Current smoking |  | P=0.609 | P=0.92 |
| No | 157/943 (17%) | 1 | 1 |
| Yes | 11/57 (19%) | 1.20 [0.61-2.36] | 1.04 [0.49-2.17] |
| **Clinical and comorbidities** |  | | |
| Body mass index (kg/m2) |  | P=0.938 | P=0.82 |
| <25 | 104/630 (17%) | 1 | 1 |
| 25-29.99 | 39/234 (17%) | 1.01 [0.68-1.51] | 0.88 [0.58-1.36] |
| ≥30 | 24/135 (18%) | 1.09 [0.67-1.78] | 1.03 [0.61-1.74] |
| Hypertension^3^ |  | P=0.440 | P=0.54 |
| Absent | 145/885 (16%) | 1 | 1 |
| Present | 22/114 (19%) | 1.22 [0.74-2.01] | 1.18 [0.70-1.98] |
| Diabetes Mellitus^4^ |  | P=0.163 | P=0.27 |
| Absent | 159/969 (16%) | 1 | 1 |
| Present | 8/30 (27%) | 1.85 [0.81-4.24] | 1.65 [0.70-3.91] |
| Chronic Kidney Disease^5^ |  | P=0.104 | P=0.14 |
| Absent | 164/961 (17%) | 1 | 1 |
| Present | 3/38 (8%) | 0.42 [0.13-1.37] | 0.44 [0.13-1.48] |
| Probable depression^6^ |  | P=0.018 | P=0.007 |
| Absent | 140/888 (16%) | 1 | 1 |
| Present | 28/112 (25%) | **1.78 [1.12-2.83]** | **1.99 [1.23-3.20]** |
| HIV status^7^ |  | P<0.001 | P=0.002 |
| PWoH | 104/500 (21%) | 1 | 1 |
| PLWH | 64/500 (13%) | **0.56 [0.40-0.89]** | **0.57 [0.40-0.81]** |

^1^Sociodemographic factors are adjusted for age group (30-39, 40-49, ≥50 years), sex and marital status. Behavioral factors are adjusted for age group, sex, marital status and alcohol use. Clinical factors and comorbidities are adjusted for age group, sex, marital status, alcohol use, probable depression, and HIV status. ^2^A composite score combining ownership of four household assets (television, refrigerator, motorcycle, and car) and presence of tap water and electricity, each contributing a 1-point. ^3^Patient Health Questionnaire (PHQ-9) score of ≥10. ^4^ Clinic Systolic blood pressure ≥140 or diastolic blood pressure ≥90. ^5^ Fasting blood glucose ≥7.0 mmol/L. ^6^ Estimated Glomerular Filtration Rate (eGFR) < 60 mL/min/1.73 m^2^ . ^7^PLWH: People Living with HIV, PWoH: People without HIV.

**Supplementary table 4.** Factors associated with excessive daytime sleepiness, stratified by HIV status^1^

|  | **Adjusted odds ratio [95% CI]** ^2^ | | **p-value for interaction**^1^ |
| --- | --- | --- | --- |
|  | **PWoH** | **PLWH** |  |
| **Sociodemographic** |  |  |  |
| Age category (years) |  |  | 0.171 |
| 30 - <40 | 1 | 1 |  |
| 40 - <50 | 1.53 (0.91 -2.57 ) | 0.69 (0.35 -1.36 ) |  |
| 50 and above | 1.72 (0.96 -3.05 ) | 1.27 (0.65 -2.50 ) |  |
| Sex |  |  | 0.225 |
| Females | 1 | 1 |  |
| Males | 0.79 (0.48 -1.28 ) | 1.24 (0.70 -2.20 ) |  |
| Marital status |  |  | 0.587 |
| Divorced/ Widowed/ Single | 1 | 1 |  |
| Married/cohabiting | 1.59 (0.94 -2.67 ) | 1.29 (0.75 -2.22 ) |  |
| **Behavioral** |  |  |  |
| Alcohol use |  |  | 0.173 |
| No | 1 | 1 |  |
| Yes | 1.27 (0.79 -2.04 ) | 2.09 (1.21 -3.60 ) |  |
| **Clinical and comorbidities** |  |  |  |
| Probable depression^3^ |  |  | 0.032 |
| Absent | 1 | 1 |  |
| Present | 1.16 (0.57 -2.39 ) | 3.30 (1.73 -6.28 ) |  |

PLWH: People Living with HIV, PWoH: People without HIV

^1^Stratified estimates are obtained by fitting an interaction term between HIV status and each covariate, with p-values obtained by comparing models with and without the interaction term. ^2^Sociodemographic factors are adjusted for age group (30-39, 40-49, ≥50 years), sex and marital status. Behavioral factors are adjusted for age group, sex, marital status and alcohol use. Clinical factors and comorbidities are adjusted for age group, sex, marital status, alcohol use and probable depression. ^3^Patient Health Questionnaire (PHQ-9) score of ≥10
